# An Historical Perspective: The Second Order Neuron in the Pain Pathway

**DOI:** 10.3389/fpain.2022.845211

**Published:** 2022-03-08

**Authors:** Andrew J. Todd

**Affiliations:** Institute of Neuroscience and Psychology, College of Medical, Veterinary and Life Sciences, University of Glasgow, Glasgow, United Kingdom

**Keywords:** spinal cord, dorsal horn, interneuron, projection neuron, pain

## Introduction

The dorsal horn of the spinal cord is the main site of termination for primary afferent axons that convey somatosensory information from the trunk and limbs. Many, if not all, of the nerve cells within the dorsal horn receive direct synaptic input from these primary afferents, and can therefore be described as “second order neurons”. Large-diameter myelinated (Aβ) afferents, which function as low-threshold mechanorceptors, send collateral branches directly to the brain, forming the first part of the dorsal column-medial lemniscus pathway. However, fine myelinated (Aδ) and unmyelinated (C) afferents, many of which function as nociceptors, terminate exclusively in the dorsal horn, and this is therefore the site of the first synapse in pathways that underlie the perception of pain. It has long been realized that the dorsal horn neurons are potential targets for therapies aimed at treating pain, and it is also clear that pathological changes occurring in this region play an important part in chronic pain states. Because of this, there have been numerous studies aimed at defining the neuronal organization and circuitry of the dorsal horn. The aim of this review is to highlight some of the key findings that have led to our current understanding of this region. To indicate the historical context of these findings and the contingent flow of events, I have arbitrarily assigned these studies to four time periods.

## Basic Organization of the Dorsal Horn: From Rolando to Rexed

Early anatomical studies identified certain specific regions within the dorsal horn. For example in 1824 Rolando (1) described a translucent zone in the superficial part, which he named the substantia gelatinosa, and subsequent studies revealed that the translucent appearance results from the lack of myelin in this region. The more ventral parts of the dorsal horn were referred to as the nucleus proprius and the neck, while the thin dorsalmost part was known as the marginal layer. However, in 1952, Rexed (2), working at the Karolinska Institute, was able to reveal a *laminar* pattern based on the size and packing density of neurons. Crucially, he recognized that this pattern extended throughout the length of the spinal cord, from cervical to sacral segments. His laminar scheme, initially developed for the cat spinal cord, has since been adapted for several other species. Rexed's pivotal work described six laminae in the dorsal horn of the lumbar and cervical enlargements, with laminae I and II corresponding to the marginal layer and substantia gelatinosa, respectively. Although this scheme was based purely on anatomical features (the size and packing density of neurons), it has turned out to be extremely useful, providing a basic map upon which more detailed elements have been overlaid. These include the terminations of different classes of primary afferents, and the distribution of neurons with specific cellular response profiles, as will be discussed below.

In other regions of the central nervous system, investigation of cell morphology provided important insight into neuronal organization, and subsequently allowed the identification of functional circuits. The earliest morphological studies of dorsal horn neurons were based on the Golgi technique, in which a small proportion of the cells are stained in their entirety, allowing anatomical reconstruction. For example, Ramon y Cajal (3) described two types of cell that differed in size and dendritic geometry in the region corresponding to laminae I-II (superficial dorsal horn, SDH): “limitrophe” (border) cells, located in the most superficial part of this region, and “central” cells found throughout the substantia gelatinosa.

## The 1960s and 70s: Early Insights Into Dorsal Horn Circuitry

**Figure 1:**
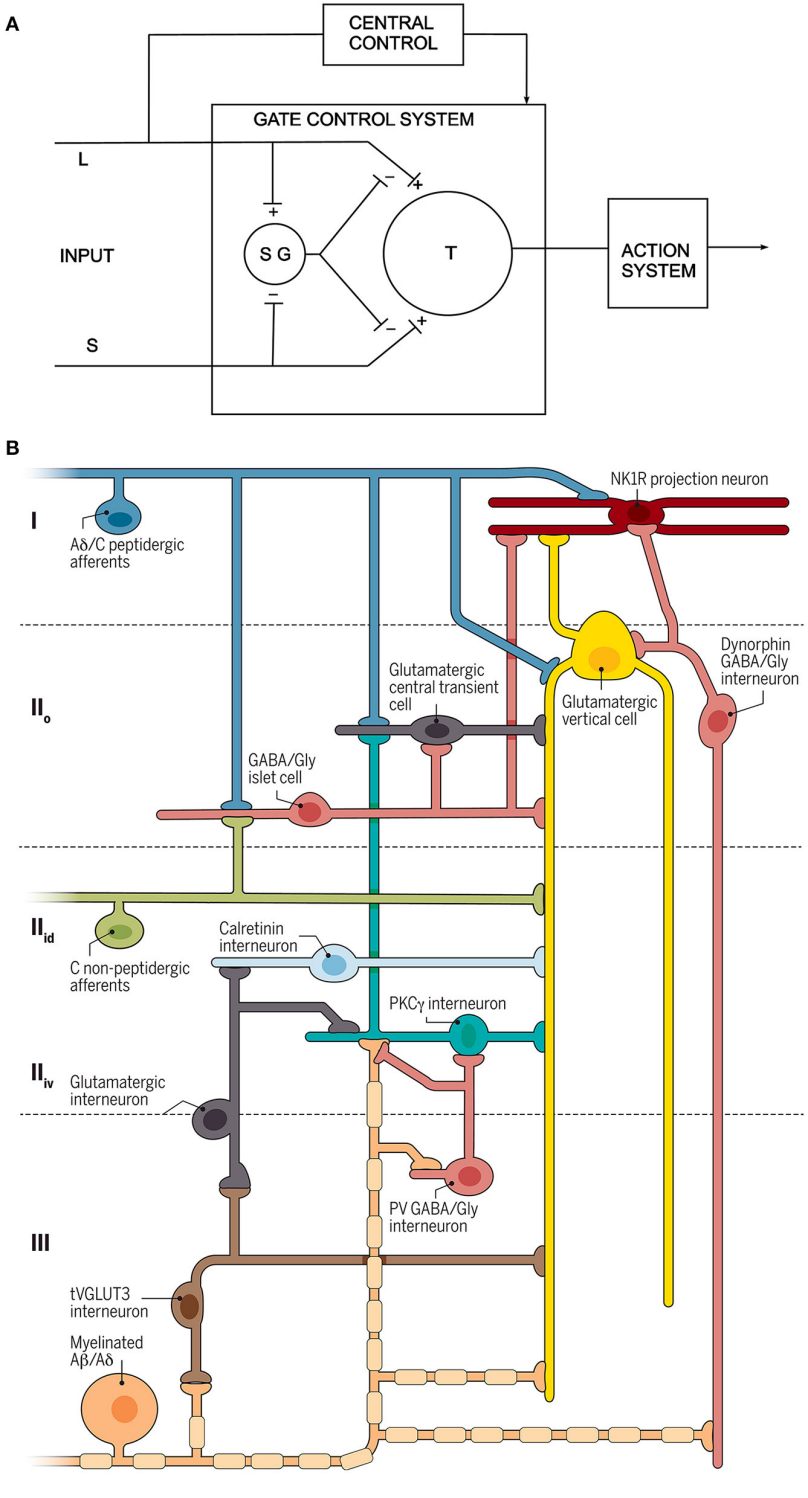
Circuit diagrams. **(A)** The circuit proposed by Melzack and Wall. L and S refer to large and small diameter primary afferents, SG is a neuron in the substantia gelatinosa (lamina II) and T is the “first central transmission cell” in the spinal cord. Modified from reference (4). **(B)** A recently proposed circuit for modulation and transmission of nociceptive and low-threshold afferent input (5). Three different types of primary afferent input are shown to the left, and the output is represented by a lamina I projection neuron that expresses the neurokinin 1 receptor (NK1R). Several different classes of interneuron are indicated. Reproduced with permission from reference (5).

This period saw the publication of the Gate Theory of Pain (4), which was the first attempt to define a neuronal circuit for somatosensory processing at the spinal cord level (Figure 1A). Other important advances included mapping of the input from different primary afferent populations, and of the cells of origin of the various ascending pathways. There was further progress in attempts to define connectivity by characterizing neuronal populations within the dorsal horn, both in terms of their morphology and their physiological properties.

Early electrophysiological studies aimed to determine the receptive field properties of dorsal horn neurons, and were mainly performed in decerebrate cats. However, they were limited by the difficulty in recording extracellular action potentials from the predominantly small neurons in laminae I-III. In 1967 Patrick Wall at University College London (UCL) (7) reported a progressive change in response properties when moving from lamina IV, where cells were generally activated by innocuous skin stimulation, to lamina V, where they could be driven by both innocuous and noxious stimuli. Cells of the latter type became known as “wide dynamic range” (WDR) or “convergent” neurons, and this response profile was seen as being typical of cells in lamina V. While examining the responses of the WDR neurons Wall, together with Mendell described a form of plasticity in which C fiber input led to a progressive increase in discharge frequency (8). This phenomenon, which they referred to as “*windup*” (reviewed elsewhere in this Frontiers series) is thought to provide an amplification of nociceptive input. In 1970, Christensen and Perl (9) obtained the first recordings from cells in lamina I, and found that these neurons could respond to noxious mechanical stimuli, and/or to thermal stimuli in either noxious or innocuous temperature ranges. By the end of the 1970s improvements in microelectrodes and advances in electronics meant that recordings could be obtained from the small neurons in laminae II and III (10, 11). These cells displayed a variety of responses, with some being activated by innocuous mechanical stimuli, others only by noxious mechanical stimuli and some responding to both types of stimulus. They also showed unexpected features, including prolonged discharges in response to brief stimuli (afterdischarges), habituation to repeated stimuli, as well as alterations in the sizes of their receptive fields (11).

Another important finding toward the end of this period was the demonstration of “diffuse noxious inhibitory controls” (DNIC) by Le Bars, Besson, and Dickenson in Paris (12). They showed that responses of WDR neurons recorded in laminae IV-V to noxious stimulation of the receptive field were suppressed by noxious stimuli applied elsewhere on the body, providing evidence for a powerful descending modulatory mechanism. DNIC is thought to underlie the phenomenon of “conditioned pain modulation”, seen in humans.

The 1970s saw the development of retrograde tracing techniques, and one of the earliest studies to use this approach to identify spinal projection neurons was published in 1975 by Trevino and Carstens at University of North Carolina (13). They showed that neurons belonging to the spinothalamic tract were concentrated in three areas: lamina I, the lateral part of laminae IV–V and a deep medial region, corresponding to laminae VII–VIII. Although earlier electrophysiological studies had identified the location of projection neurons by using antidromic activation, a major advantage of the retrograde tracing technique was that it could reveal large numbers of neurons in a single experiment. This was followed by numerous studies in various species, which mapped the cells of origin of projection cells in greater detail. Advances included identifying neurons that projected to specific regions of the thalamus, and to other brainstem structures such as the periaqueductal gray matter, the lateral parabrachial area and the reticular formation. These are described in more detail in another article in this issue.

Meanwhile anatomical studies were revealing further details about the morphology of neurons in the superficial laminae. In 1978, Gobel at the National Institute of Dental Research (14) defined several morphologically distinct classes of neurons in lamina II of the cat spinal trigeminal nucleus (the medullary homolog of the spinal dorsal horn). Among these classes, two have consistently been identified in subsequent studies: islet cells, which had dendritic and axonal arbors that were highly elongated along the rostrocaudal axis; and stalked cells (probably equivalent to Cajal's “limitrophe” cells), which had a cell body located in the dorsal part of lamina II, dendrites that passed ventrally and an axon that entered lamina I. Based purely on their morphology, Gobel speculated that islet cells were inhibitory, and stalked cells excitatory, a prediction that was subsequently confirmed with immunohistochemistry (15). An alternative view was reached by Beal and Cooper at Wayne State University (16), based on their Golgi studies of laminae II-III in the monkey. They also observed cells similar to those described by Gobel, but concluded that neurons in this region were so diverse that they defied classification. In 1979, Light et al. (17) achieved the first intracellular recording from superficial dorsal horn neurons *in vivo*, thus allowing comparison of the morphology of individual cells with their responses to natural stimuli. However, in agreement with Beal and Cooper, they were unable to recognize distinct morphological classes. Nonetheless, in the following year Bennett et al. (18), using a similar approach, reported that stalked and islet cells located in the dorsal part of lamina II had nociceptive-specific or WDR receptive fields, whereas islet cells in the ventral part of lamina II responded only to innocuous mechanical stimuli.

Further details also emerged concerning the “first order” (primary afferent) neurons, and this was key to understanding the receptive field properties of neurons in different laminae. While earlier studies had shown the basic arrangement of the input, detailed termination patterns for Aβ and Aδ afferents were revealed following intra-axonal recording *in vivo* (19, 20). These studies revealed that different types of Aβ cutaneous afferent had characteristic axonal arbors within the deep dorsal horn (laminae III-VI), Aδ hair afferents terminated on either side of the lamina II-III border, and Aδ nociceptors innervated lamina I, with some extension into lamina V.

## 1980–2010: Consolidation of Knowledge

During this period there was steady progress on many different fronts, and it is only possible to select a few representative examples.

Anatomical and electrophysiological studies provided further insight into the organization of projection neurons. Menetrey et al. (21) reported that those located in the deep dorsal horn had relatively large receptive fields, and included cells with low-threshold, nociceptive and WDR responses. In contrast, a later study by Bester et al. (22) showed that projection cells in lamina I had much smaller receptive fields, and invariably responded to both noxious thermal and mechanical stimuli, with some also showing moderate responses to innocuous mechanical stimuli. A key observation during this period was that projection cells only accounted for a very small proportion (likely ~1%) of the neurons throughout the spinal gray matter, with the vast majority being interneurons (23). The finding that most of the projection neurons in laminae I and III expressed the neurokinin 1 receptor (NK1r) meant that immunohistochemical staining for the receptor could be used to reveal dendritic trees of these cells. This approach was used to show that they receive a high density of synapses from peptidergic primary afferent nociceptors (24), thus forming the most direct (monosynaptic) route through which nociceptive information reaches the brain. Ablation of NK1r-expressing cells by intrathecal administration of the ligand (substance P) conjugated to saporin was found to result in a reduction of neuropathic and inflammatory hypersensitivity but no change in acute pain thresholds (25), suggesting that lamina I projection cells may be more important for pathological pain states than for acute pain. The debate about the relative contribution of deep (laminae IV–V) vs. superficial (lamina I) projection neurons to different aspects of pain perception continues to this day.

In 1983 Clifford Woolf, at UCL, following on from the discovery of windup by Mendell and Wall (8), provided the first evidence for a central component to the sensitization that results from peripheral tissue injury (26). He showed that after a thermal injury to the hindpaw of decerebrate rats, there was a reduction in the threshold for eliciting flexion withdrawal reflexes. By recording from motoneuron axons and stimulating the sural nerve in decerebrate animals, he was then able to show that the peripheral injury resulted in an increased response of motoneurons to electrical stimulation of Aδ and C cutaneous afferents. His discovery of central sensitization, was a major advance, providing the first direct evidence that synaptic plasticity in the spinal cord contributed to pathological pain.

Intracellular recording and labeling of individual C fibers, which was achieved during this period, revealed their central arbors in laminae I-II (27). However, much of what we know about C fiber termination within the dorsal horn came from studies using neurochemical markers such as neuropeptides, or binding of the lectin IB4. In particular, it became apparent that there were two broad classes of C fiber nociceptor: peptidergic and non-peptidergic, which differed in their dependence on trophic factors and in their termination zones within the superficial dorsal horn (28).

Further insight into the role of spinal cord inhibition was provided in 1989 by Yaksh at the Mayo Clinic (29), who showed that intrathecal administration of GABA_A_ and glycine receptor antagonists in awake animals resulted in hypersensitivity to tactile stimuli. This led to the suggestion that low-threshold cutaneous input to the deep dorsal horn (laminae III–VI) can gain access to pathways that process pain-relevant information, and that this is normally suppressed by local GABAergic/glycinergic inhibition. An immunohistochemical study published in the following year suggested that around one-third of neurons in laminae I–III of the dorsal horn are inhibitory interneurons, and that many of these use GABA and glycine as co-transmitters (30). By exclusion, it was assumed that the remaining neurons were excitatory, glutamatergic cells, but this could not be directly demonstrated until the identification of the vesicular glutamate transporters more than 10 years later (31).

A crucial finding during this period was the discovery by Hunt et al. at University College London that the transcription factor Fos could be used as a marker of neuronal activity (32). Later work by Ji et al. at Harvard Medical School revealed that phosphorylation of extracellular signal-regulated kinases provided an alternative activity marker (33). These observations allowed immunohistochemical identification of cells that responded to a variety of noxious stimuli, and since these approaches could readily be combined with other anatomical methods (such as retrograde tracing) this led to important insights into the functional roles of different neuronal populations.

A major technical advance was the development of *ex vivo* preparations (in particular spinal cord slices), and the applicatoin of whole-cell patch-clamp recording, for example by Yoshimura and Nishi at Kurume University (34). This allowed far more detailed investigation of individual neurons, including the demonstration of their primary afferent input, as well as characterization of the expression of receptors and ion channels. While initially performed on unidentified neurons, this technique was subsequently adapted to allow targeted recording from specific genetically-identified neuronal populations, for example labeled with fluorescent proteins.

A key question that emerged from anatomical and electrophysiological studies during this period was how to make sense of the considerable heterogeneity of dorsal horn interneurons (35). Whole-cell recording was particularly helpful here, as subsequent morphological reconstruction of axonal and dendritic arbors could be combined with information on primary afferent input and action potential firing patterns (reflecting ion channel expression). Grudt and Perl at the University of North Carolina (36) used this approach to update earlier attempts at morphological classification. They defined four main classes of lamina II neuron, including the islet and stalked cells identified by Gobel, although the latter were renamed vertical cells. Their other two populations consisted of radial cells (with short highly-branched dendrites) and central cells, with rostrocaudally-elongated dendrites that were less extensive than those of islet cells. By extending this approach to include identification of transmitter phenotype through the detection of vesicular neurotransmitter transporters, Yasaka et al. at the University of Glasgow (37) were able to confirm that islet cells were inhibitory and that stalked/vertical cells were excitatory. They also showed that the radial cells identified by Grudt and Perl were excitatory, whereas those defined as central cells could be either excitatory or inhibitory, indicating that this morphological class did not correspond to a single functional population. Another theme that emerged during this period, was that the complex neurochemistry of the superficial dorsal horn could be used to provide an alternative approach for neuronal classification. In particular, several neuropeptides that were present in this region were found to be associated with either excitatory or inhibitory interneurons (38), and subsequent work has shown that these can be used to define specific functional populations (39). A particular advantage of this approach has been that it can be combined with mouse genetics (see below) to allow targeting of neuronal populations for anatomical, electrophysiological and behavioral studies.

## 2010 to the Present: The Age of Mouse Genetics

The last decade has seen a revolution in our understanding of spinal sensory processing, largely as a result of advances in mouse genetics. A study by Duan et al. (40) was one of the first to examine the roles of genetically-defined interneuron populations on pain behavior. Somatostatin is expressed by the majority of SDH excitatory interneurons, but apparently not by projection neurons, and they found that ablating somatostatin-expressing cells greatly reduced responses to noxious mechanical (but not thermal) stimuli, indicating that excitatory interneurons form an essential part of the circuitry that underlies mechanical pain. Although earlier neurochemical studies had shed light on the organization of neurons and circuits in the SDH, the deeper laminae remained *terra incognita*, due to the lack of obvious markers. By screening for genes that were selectively expressed in populations of neurons in the region extending from the inner part of lamina II to lamina IV, Abraira et al. (41) were able to identify seven types of excitatory interneuron and four types of inhibitory interneuron. These cells showed distinct morphological and electrophysiological properties, as well as characteristic patterns of input from the low-threshold mechanoreceptive afferents that terminate in this zone.

Classification of neuronal populations is essential for our understanding of the circuits that process somatosensory information, but until recently neurochemical classification schemes had depended on the identification of potential markers, and this is inevitably rather hit-and-miss. Single cell RNA sequencing studies have provided a far more systematic approach, by assigning all of the sampled cells to clusters, based on patterns of gene expression. For example, Häring et al. at the Karolinksa (6) identified 15 clusters each for excitatory and inhibitory dorsal horn neurons, and then used *in situ* hybridization to define their laminar locations. Reassuringly, there was reasonably good agreement with previous neurochemical classification schemes, but additional populations were revealed.

## Concluding Comments

As reviewed here, our understanding of the complex role played by second order dorsal horn neurons has evolved from the anatomical insights acquired during the 19th and early 20th centuries, through the electrophysiological studies in the latter part of the 20th century that began to reveal the behavior of these cells. The last few years have seen dramatic advances in our understanding of the organization of the neural circuits engaged by the second order neurons, largely due to the recognition that there are in fact many different types of “second order neuron”, and that these can be distinguished based on their neurochemistry. This, in turn, has led to the recognition that the functional connections between primary afferents, descending axons, dorsal horn interneurons and projection cells are highly organized, resulting in intricate synaptic circuits through which sensory information is transmitted and modulated. This evolution of knowledge can be seen by comparing a recent circuit diagram (5) with the one originally proposed by Melzack and Wall (4) (Figure 1). Sadly, space prevented mention of all those who have contributed so measurably to these insights.

## Author Contributions

AJT performed the literature search and wrote this opinion article.

## Funding

Work in the author's laboratory was funded by grants from the Medical Research Council (grant number MR/S002987/1) and the Wellcome Trust (grant numbers 102645/Z/13/Z and 219433/Z/19/Z).

## Conflict of Interest

The author declares that the research was conducted in the absence of any commercial or financial relationships that could be construed as a potential conflict of interest.

## Publisher's Note

All claims expressed in this article are solely those of the authors and do not necessarily represent those of their affiliated organizations, or those of the publisher, the editors and the reviewers. Any product that may be evaluated in this article, or claim that may be made by its manufacturer, is not guaranteed or endorsed by the publisher.
